# Editorial: Skin Blistering Diseases

**DOI:** 10.3389/fmed.2019.00060

**Published:** 2019-04-02

**Authors:** Cristina Has, Kyle T. Amber, Dedee F. Murrell, Philippe Musette, Ralf J. Ludwig

**Affiliations:** ^1^Department of Dermatology, Faculty of Medicine, University of Freiburg, Freiburg, Germany; ^2^Department of Dermatology, University of Illinois at Chicago, Chicago, IL, United States; ^3^Department of Dermatology, St George Hospital, University of New South Wales, Sydney, NSW, Australia; ^4^Department of Dermatology, Rouen University Hospital, Rouen, France; ^5^Lübeck Institute of Experimental Dermatology and Center for Research on Inflammation of the Skin, University of Lübeck, Lübeck, Germany

**Keywords:** skin, autoimmunity, hereditary diseases, pemphigoid, pemphigus, epidermolysis bollosa

Skin blistering is commonly caused by mechanical, physical, or infectious insults. Less often, mutations of structural components of the skin (1) or autoimmunity (2) directed against those structural components (3) lead to skin blistering. Albeit among the less frequent causes of skin blistering, understanding of the pathomechanisms of hereditary and autoimmune skin blistering has provided detailed insights into cutaneous biology and (auto)immunity. More specifically, in recent years, genetics, definition of autoantigens, model systems, and clinical research have led to a tremendous improvement for both diagnosis and treatment for patients suffering from skin blistering diseases. This is well-reflected by the article within the Research Topic Skin Blistering Diseases: Regarding genetics Vodo et al. herein review the genetics of pemphigus vulgaris (PV). As for many autoimmune diseases, the strongest association is observed for the HLA-locus. Thus far, 3 genome-wide association studies (GWAS) for PV, have found ST18 and TAP2 to be associated with PV (4–6). PV is a potentially fatal autoimmune blistering disease, characterized, and caused by autoantibodies targeting epithelial desmosomal antigens. The autoantibodies in PV are mainly directed against desmoglein (Dsg) 3 and −1. However, as highlighted by Sinha and Sajda in the Research Topic Skin Blistering Diseases, the anti-Dsg1/3 immune response does not explain the disease heterogeneity in PV. Indeed, the advance of technology, such as protein microarrays, has identified a number of additional autoantibodies in PV patients (7). Based on these observations, they here propose a super-compensation hypothesis, whereby the mixture, specificity and pathogenicity of PV-specific autoantibodies determine the clinical disease presentation. Whereas in pemphigus, and other autoimmune skin blistering diseases, genetics is mostly used to obtain insights into disease pathogenesis, genetic research in hereditary blistering diseases is far advanced. As reported by Condrat et al. in the Research Topic Skin Blistering Diseases, allelic heterogeneity and mutation status are at the verge of being employed for molecular-targeted, personalized treatment for patients with junctional epidermolysis bullosa (JEB), caused by reduced dermal-epidermal adhesion due to deficiencies of specific hemi-desmosomal adhesion proteins, such as type XVII collagen or laminin-332 (8).

Insights into the pathogenesis of skin blistering diseases have been reviewed in detail elsewhere (9–11). Within the Research Topic Skin Blistering Diseases, the role of eosinophils in bullous pemphigoid (BP) has been highlighted. BP is the most common subepidermal autoimmune skin blistering disease (12), with a variable clinical presentation, caused by autoantibodies targeting BP180 (9) and/or BP230 (13, 14). The contribution of eosinophils, albeit constituting the majority of cells observed in the dermal infiltrate (15), to BP pathogenesis remains uncertain. In their review, Amber et al. review the evidence for a pro-inflammatory role of eosinophils in BP, demonstrating pathways both dependent and independent of IgE autoantibodies targeting the major autoantigen (BP180) (16).

During the past decades, the improved diagnosis of autoimmune skin blistering has partially contributed to the observed increase in the incidence of those diseases, especially BP. With the availability of commercial test systems for the diagnosis of epidermolysis bullosa acquisita (EBA), in line with an increased awareness for this rare autoimmune skin blistering disease (17), we also expect that EBA will be diagnosed more often. Two original articles in the Research Topic Skin Blistering Diseases addressed the significance of indirect immunofluorescence (IF) microscopy for the diagnosis of autoimmune skin blistering. Kridin and Bergman demonstrate that the predictive value of negative finding in IF microscopy using monkey esophagus as a substrate is reliable diagnostic test to exclude the diagnosis of pemphigus. In mucous membrane pemphigoid (MMP), an autoimmune blistering disease with predominant mucosal involvement, caused by a variety of autoantibodies (15), diagnosis is often difficult due to non-specific destruction of the mucosal biopsies and due to the low titers of circulating autoantibodies. By performing direct IF microscopy of peri-lesional mucosal biopsies from MMP patients, Kamaguchi et al. demonstrated a better sensitivity and specificity compared to H&E staining and serology. In addition to laboratory testing, careful clinical examination will provide important clues for diagnosis. In the article by Pietkiewicz et al. the frequency of nail involvement in pemphigus patients was determined, as this had been published on a case report basis and prospective data had been not available. Collectively, they demonstrated a low frequency of nail involvement in pemphigus.

Based on these insights into disease pathogenesis, many novel treatments have emerged, which were jointly presented and discussed among patients, clinicians, and researchers at the 5th International Pemphigus and Pemphigoid Foundation Scientific Conference held in Orlando, Florida, on May 15–16, 2018. In the flanking Perspectives Article, Lee et al. have summarized the emerging treatments for pemphigus and pemphigoid diseases. As highlighted by 2 case reports, one focusing on the treatment of paraneoplastic MMP, and one on the treatment of pemphigus with rituximab, current treatment still relies on systemic immunosuppression, which causes significant patient morbidity and mortality. In addition, long-term immunosuppression also fails to induce remission, especially in EBA (18–20). Hence, novel treatments are urgently needed to meet this unmet medical need (21). With the advent of consensus definitions (22–24) and the development and validation of objective (25, 26) and subjective (27) scoring systems to measure treatment responses objectively, we have entered the era of sponsored clinical trials of new treatments for these orphan blistering diseases.

As highlighted by Bech et al. the treatment of autoimmune skin blistering diseases, is becoming more challenging due to the observed co-morbidity. They highlight that in BP the high incidence of cardiovascular and neurological co-morbidity must be considered when selecting treatments for BP. Use of high doses of corticosteroids, the backbone of BP treatment, may aggravate cardiovascular co-morbidity, and be at least partially responsible for the increased cardiovascular mortality in BP patients. Furthermore, co-occurrence of chronic inflammatory diseases, such as psoriasis and autoimmune skin blistering diseases, need to be taken into consideration when selecting treatments. However, the Research Topic Skin Blistering Diseases Kridin et al. demonstrate that the anecdotal association of pemphigus with thyroid diseases can only be partially confirmed in a large cohort of pemphigus patients. The authors show that Hashimoto's thyroiditis is associated with pemphigus in male patients, while no association of pemphigus with Graves' disease or thyroid cancer was detected.

With the Research Topic Skin Blistering Diseases we aim to increase awareness for these diseases, present the state-of-the art diagnosis and treatment, and (maybe most importantly) stimulate further basic, translational and clinical research in the field.

## Author Contributions

All authors listed have made a substantial, direct and intellectual contribution to the work, and approved it for publication.

### Conflict of Interest Statement

RL has received honoraria and/or research grants from the following companies: Admirx, Almirall, Amryth, ArgenX, Biotest, Biogen, Euroimmun, Incyte, Immungenetics, Lilly, Novartis, UCB Pharma, Topadur, True North Therapeutics and Tx Cell. The remaining authors declare that the research was conducted in the absence of any commercial or financial relationships that could be construed as a potential conflict of interest.
